# Structural violence and the biomedical innovation system: what responsibility do universities have in ensuring access to health technologies?

**DOI:** 10.1136/bmjgh-2020-004916

**Published:** 2021-05-03

**Authors:** Sarai Keestra

**Affiliations:** 1 Amsterdam UMC, University of Amsterdam, Amsterdam, The Netherlands; 2 Global Health and Development, London School of Hygiene & Tropical Medicine, London, UK

**Keywords:** health policy, vaccines

**Summary box**

**What is already known?**

- When universities engage in inequitable technology transfer practices, by patenting and exclusively licensing biomedical innovations in ways enabling pricing monopoly formation, they contribute to harmful barriers to access to medicines and other health technologies for millions of people.

**What are the new findings?**

- Universities are contributing to the violation of the social and economic rights of those unable to access novel health technologies by unquestioningly engaging in a biomedical innovation system that relies on profit-driven commercialisation of knowledge generated with public funding, and this should be considered structural violence.
- Applying the lens of structural violence can help reframe the responsibilities of universities in the access to medicines debate by: (1) highlighting universities’ position in the structures of the biomedical innovation system, (2) bringing attention to the systematic inequities in knowledge dissemination and how this can result in differential health outcomes when particular groups are denied timely access to health innovations and (3) reconsidering the role of universities’ technology transfer practices in sustaining the unequal structures and power relations inherent to the biomedical innovation system.

**What do the new findings imply?**

- Universities can apply equitable technology transfer practices such as non-exclusive, royalty-free licensing of biomedical innovations to promote access to health technologies, ensuring that all members of the global public can enjoy the fruits of scientific progress.

## Inequitable technology transfer practices as structural violence

Universities intend to create knowledge that serves the needs of the public, yet this does not always happen in practice. By engaging in inequitable technology transfer practices, such as the exclusive licensing of a novel health technology to a private company or a spin-off, universities enable the downstream formation of pricing monopolies that limit affordable access to health technologies.^1 2^ The WHO defines health technologies as ‘the application of organized knowledge and skills in the form of devices, medicines, vaccines, procedures and systems developed to solve a health problem and improve quality of lives’.^3^ Worldwide nearly 2 billion people lack access to essential medicines and 100 million people are pushed into extreme poverty annually due to their inability to pay for their healthcare expenditures.^4 5^ While revenues from licensing contribute less than 4% to universities’ income, since the introduction of the Bayh-Dole Act (1980) the pursuit of intellectual property (IP) rights has become common practice at higher education institutions.^2 6^ Universities occupy an unique position in the innovation ecosystem between upstream research and development (R&D), which is often publicly funded, and downstream commercialisation by the private sector. Universities’ decisions on the conditions of technology transfer are an opportunity to resist the status quo of a system that is causing a global tragedy of preventable deaths by prioritising profits over health.

Structural violence, a concept from peace research introduced into the global health discourse by physician–anthropologist Paul Farmer, describes how institutional structures as well as social, historical and economical inequities determine whose human rights are violated by being denied access to the fruits of scientific and medical progress.^7^ According to Farmer these systematic inequalities ‘are structural because they are embedded in the political and economic organization of our social world’ and ‘violent because they cause injury to people’.^8^ Whenever novel health technologies become available to only a segment of global society, the health gap between the affluent and the poor widens. Meanwhile the number of fatalities that can be completely prevented with health technologies already available to the fortunate, which Farmer has previously called ‘stupid deaths’, increases.^7^ Approximately 50 000 people die prematurely each day from curable medical afflictions.^9^ These are victims of structural violence, as inequalities in access to healthcare services and affordable medicines is intimately linked to inequalities of power, perpetrated by our system of IP, as well as social and economic inequities that determine ‘who suffers and who is shielded’.^7^ If these injustices are not constantly questioned and resisted, existing inequities in the biomedical innovation system are permitted to become further entrenched and normalised.^7^ By taking part in inequitable technology transfer practices that perpetuate the existence of an inequitable IP system, universities are complicit in violations of the human rights ‘to a standard of living adequate for the health and well-being of himself and of his family, including (…) medical care’ (art.25) and ‘“to share in scientific advancement and its benefits’ (art.27).^10^

Harm does not need to be intentional for it to qualify as structural violence. At the height of the AIDS epidemic in 2001, universities held ~25% of patents on antiretroviral drugs, including lamivudine (Emory), stavudine (Yale) and enfuvirtide (Duke).^2 11^ Three million AIDS deaths were reported that year, many attributable to the lack of access to affordable treatment.^12^ The universities owning the patents of these life-saving yet unaffordable drugs are enablers of the structural violence that resulted in these deaths by contributing to the formation of pricing monopolies when they exclusively licensed their IP rights to private industry. After extensive campaigning by students, faculty members and health activists, Yale pressured Bristol Myers Squibb, the pharmaceutical company licensed with the exclusive rights to stavudine, into not enforcing its academic patent in South Africa.^13^ Consequently, the price of the patented drug decreased 30-fold without any negative consequences to the university itself,^11^ demonstrating that the academic community can play a powerful role in ensuring equitable access to health technologies.

## Equitable technology transfer as a means to challenge inequities in the biomedical innovation system

A critical awareness is needed that the current profit-driven commercialisation of science is in stark contrast with the mandate of universities as uniquely privileged spaces where research is conducted for the benefit of public well-being.^2 14^ The dissemination of university research should be rooted in the concept of global health equity, which envisions a needs-based approach to health and well-being of humanity rather than one based on economic and social privileges.^1^ To uphold these commitments to inclusive knowledge dissemination in the interest of the public, universities should ensure during the technology transfer process that the global community receives a return on investing into R&D.^1 6 15^ Indeed, pharmaceutical companies often claim that the exclusivity of patents and pricing monopolies promote risk taking in early innovation, yet universities are often the source of important publicly funded research that underlies novel health technologies.^6^ Universities therefore have the opportunity to prevent the creation of pricing monopolies and access barriers downstream, and a responsibility towards the global public who contributed to R&D funding to do so.

Universities’ technology transfer offices should critically reconsider the downstream impact of filing patents in low-income and middle-income countries (LMICs) where they might act as a barrier to access, instead allowing for generic production.^1 11^ For Yale’s stavudine during the HIV/AIDS crisis this significantly decreased the price.^11^ Additionally, universities should consider retaining the right to issue voluntary non-exclusive licenses in the case of health emergencies, such as the COVID-19 pandemic^14^, and implementing step-in rights if commercial partners are not adhering to access conditions. By attaching access-oriented clauses to contracts during the technology transfer process, universities can do their part in promoting a more just biomedical innovation system. As the pandemic has brought access issues to the forefront of our collective attention, now is the right time for universities to adopt more equitable policies on technology transfer in line with their commitments to knowledge dissemination for the benefit of the public.

## The Oxford–AstraZeneca vaccine in the context of structural violence

Before the pandemic, Oxford was only one of seven UK universities committed to the principles of social responsible licensing.^1 16^ Oxford University Innovation also set an important precedent with their statement on ‘Expedited access for COVID-19 related IP’, which announced the use of non-exclusive, royalty-free licences as the default approach for technology transfer for the duration of the pandemic.^17^ Oxford’s commitments to sell the vaccine not-for-profit for the duration of the pandemic are reflected in the affordable pricing of the Oxford–AstraZeneca COVID-19 vaccine compared with alternative options on the market.^18^

Yet, LMICs’ access to COVID-19 health technologies, including the Oxford–AstraZeneca vaccine, is currently hampered by a synergy of restrictive patents, lack of equitable technology transfer and associated knowhow, advanced market commitments by high-income countries (HICs), temporary export bans, and a dearth of funding for scale-up of local manufacturing capacity.^19^ Where LMICs have received access, some sources suggest that they are paying more than HICs, for example, South Africa is reportedly paying US$5.25 per dose for the Serum Institute produced Oxford–AstraZeneca vaccine, compared with the European Union which is allegedly paying US$3.50 to AstraZeneca directly.^18 19^ Although some of the key clinical trials for the Oxford–AstraZeneca vaccine were conducted in South Africa and Brazil,^20^ LMICs struggle to secure a vaccine supply,^18 19^ laying bare the structural inequalities in the biomedical innovation system.

Despite ensuring that it is one of the most affordable vaccines,^17 18^ at least during the pandemic, by making an exclusive deal with a pharmaceutical company like AstraZeneca, Oxford transferred the power over knowledge dissemination of a technology largely developed using government and charitable funding to the private sector.^21^ The Oxford chimpanzee adenovirus vector (ChAdOx) vaccine platform was in an advanced development stage at the time of the technology transfer, while for the BioNTech/Pfizer and Moderna vaccines the privatisation of the public knowledge base happened early in the R&D process. Given this strengthened negotiation position, Oxford could have considered alternative modes of technology transfer that promote affordable access such as non-exclusive licensing to multiple pharmaceutical companies in LMICs, or putting the IP and associated knowhow into the WHO’s COVID-19 Technology Access Pool. The technology transfer process around the ChAdOx1 nCoV-19 vaccine should therefore have been done more equitably and transparently, with Oxford retaining the power to put its commitments towards inclusive knowledge dissemination into practice to prevent inequalities in vaccine access arising, especially post-pandemic when affordable pricing conditions may no longer apply but the need for an affordable COVID-19 vaccine persists.

## Conclusion

The biomedical innovation system in its current form enables the generation of excessive private profits from novel health technologies at the expense of people’s lives, which should be considered structural violence. Universities carry responsibility for contributing to preventable deaths and suffering when they fail to challenge the status quo of a biomedical innovation system that denies access to life-saving treatments for millions. Existing inequalities are deepened if universities unquestioningly participate in a system that relies on the profit-driven commercialisation of collective knowledge. Universities, critically aware of their responsibilities towards inclusive knowledge sharing, can instead challenge the ongoing structural violence by changing their technology transfer practices in ways that promote a more equitable biomedical innovation system.
